# Updates in the Management of Richter Transformation

**DOI:** 10.3390/cancers17010095

**Published:** 2024-12-31

**Authors:** Noa Rippel, Richard Sheppard, Adam S. Kittai

**Affiliations:** Tisch Cancer Institute, Icahn School of Medicine at Mount Sinai, New York, NY 10029, USA

**Keywords:** Richter transformation, targeted therapies, CLL

## Abstract

Richter transformation (RT) is a rare albeit devastating complication of chronic lymphocytic leukemia/small lymphocytic lymphoma (CLL). RT is defined as an aggressive lymphoma, typically diffuse large B-cell lymphoma, in the setting of CLL. A clonal relationship to the preceding CLL clone is detected in the majority of RT cases and confers more aggressive clinicopathologic kinetics, resistance to standard chemoimmunotherapy regimens, and inferior survival. Taken together, these considerations precipitate a significant unmet need for novel therapeutic strategies that improve the outcomes of patients with RT. Through this review, we will explore current data on emerging regimens targeting BTK, BCL-2, CD79, CD20, PI3K, and PD-1—both as single agents and as combination therapies with or without concurrent chemoimmunotherapy. Furthermore, we will review the role of bispecific T-cell engagers, anti-CD19 chimeric antigen receptor T-cell therapies, and hematopoietic stem cell transplantation in RT. To guide therapeutic decision-making, we will outline an algorithmic approach to the management of RT, with particular emphasis on prioritization of clinical trial enrollment and utilization of an ever-evolving array of novel therapies.

## Simple Summary

Richter transformation (RT) describes a rare progression of chronic lymphocytic leukemia (CLL) to an aggressive lymphoma. When RT directly evolves from the preceding CLL, termed clonally related RT, as is most often the case, patients are susceptible to particularly poor outcomes, with poor responses to traditional chemoimmunotherapy and short survival. As such, there is an urgent need to expand our treatment arsenal for RT. In recent years, clinical trials have investigated a multitude of novel drug combinations to treat RT. These include agents that block lymphoma pro-survival pathways, antibodies that facilitate tumor cell recognition by innate T-cells, and the infusion of T-cells that are genetically altered to target lymphoma. This review aims to provide a comprehensive update on various novel RT treatment strategies under investigation that have demonstrated promising efficacy and safety outcomes.

## 1. Introduction

Richter transformation (RT) is defined as a histologic evolution from chronic lymphocytic leukemia/small lymphocytic lymphoma (CLL) to a high-grade lymphoma [1]. This phenomenon was named after Maurice Richter, who had first described a transformation of CLL to an aggressive neoplasm in 1928 [2]. Of the RT subtypes, diffuse large B-cell lymphoma (DLBCL-RT) is most common, constituting approximately 95% of cases [3,4,5,6]. Among other histologic subtypes, the Hodgkin variant (HV-RT) is the second most common, constituting 0.5–5% of cases [7]. Herein, RT will refer to DLBCL-RT.

Approximately 2–10% of patients with CLL progress to RT within a median time of 2 to 4 years from CLL diagnosis, with incidence influenced by clinicopathologic disease features and both the depth and duration of response to prior therapies [8,9]. RT represents true evolution from a preceding CLL clone in 70–80% of cases, with a clonal relationship conferring a more aggressive disease course, poor responses to chemoimmunotherapy (CIT) and novel agents, and markedly worse survival outcomes [10].

The management of RT remains an area of markedly unmet need. Its heterogeneous presentation, adverse disease kinetics, and poor response to common lymphoma-directed therapies contribute to an absence of a clear consensus on guideline-directed care. Herein, we provide a review of the pathogenesis and diagnostic workup, prognostic markers that predict survival, followed by a detailed examination of emerging single-agent and combination modalities that have demonstrated encouraging outcomes for RT.

## 2. Pathogenesis

The underlying mechanisms driving RT are multifactorial, involving a complex interplay of genetic alterations, epigenetic modifications, and interactions with the tumor microenvironment. Genetic alterations play a prominent role, with early studies identifying key mutations and chromosomal abnormalities that contribute to the clonal evolution seen in RT [10,11]. *TP53*, *NOTCH1*, *MYC*, and *BIRC3* have been observed with greater frequency in RT than in typical CLL progression, suggesting that these alterations may drive the aggressive phenotype and influence poor patient prognosis [6,12].

Recent data also highlight the role of epigenetic dysregulation, particularly involving methylation patterns and histone modifications as contributors to the aggressive biology of RT [13]. Additionally, the tumor microenvironment, including interactions with surrounding stromal cells and immune cells, appears to facilitate transformation through cytokine and receptor-mediated signaling pathways [14]. The interplay of these factors, combined with the potential selective pressure from CLL therapies, appears to create a landscape conducive to transformation [15].

## 3. Diagnosis

### 3.1. Clinical Presentation

**Figure 1:**
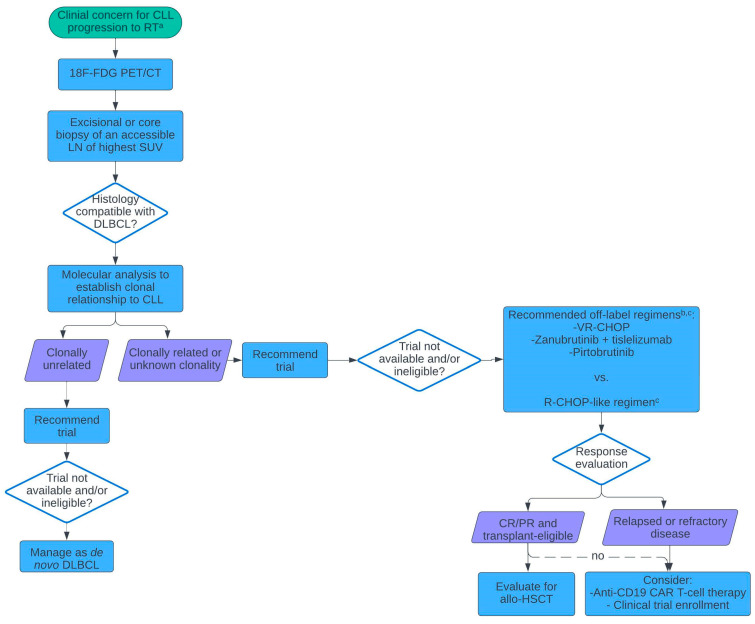
Suggested algorithm for a diagnostic and therapeutic approach to RT. ^a^: based on new/worsening constitutional symptoms, physical decline, rapidly enlarging lymphadenopathy, extra nodal disease, progressive cytopenias, and a sudden/unexpected rise in LDH. ^b^: To be determined based on patient-specific factors and institutional practice. ^c^: as per the National Comprehensive Cancer Network-suggested CIT regimens for clonally related or unknown clonal status RT [17]. Allo-HSCT: allogeneic hematopoietic stem cell transplant; CAR-T: chimeric antigen receptor T-cell; CIT: chemoimmunotherapy; CLL: chronic lymphocytic leukemia; CR: complete remission; DLBCL: diffuse large B-cell lymphoma; PR: partial response; RT: Richter transformation; VR-CHOP: venetoclax, rituximab, cyclophosphamide, doxorubicin, vincristine, prednisone; 18F-FDG PET/CT: fluorodeoxyglucose fluorine 18 positron emission tomography with computer tomography.

While RT may occur at any time point for a patient with CLL, it is most commonly diagnosed within the first 2 years of starting CLL-directed therapy [3]. In a retrospective analysis, we previously identified clinicopathologic characteristics associated with the development of RT at time of disease progression among patients with CLL who were treated with ibrutinib [16]. Within a median follow-up time of 44.5 months from treatment initiation, 179 of 559 experienced disease progression. Of these, 54 developed RT after a median follow-up of 20.8 months from progression. Independent prognostic factors for transformation to RT included progression on treatment (HR 4.01 [1.60–10.00], *p* = 0.003), lymphadenopathy without lymphocytosis (HR 2.88 [1.15–7.20], *p* = 0.02), and a higher LDH level (for 2-fold increase, HR 1.80 [1.33–2.43], *p* = 0.0001) [16]. These data highlight when clinicians should have a suspicion for RT (Figure 1).

### 3.2. Diagnostic Tools

Imaging is central for identifying sites suggestive of RT involvement and to guide subsequent tissue biopsy. Fluorodeoxyglucose fluorine 18 positron emission tomography with computed tomography (18F-FDG PET/CT) is the modality of choice, with an SUVmax of ≥5 having positive and negative predictive values of 51–53% and 94–97%, respectively, for the diagnosis of RT [18,19]. With a greater SUVmax cutoff of >10, sensitivity and specificity have been demonstrated to be 91% and 95%, respectively [20]. Oftentimes, however, SUVmax will be much higher with RT; in a study of 332 patients with CLL, RT (n = 95) was associated with a median SUVmax of 17.6, compared to 3.7 and 6.8 in histologically indolent vs. aggressive CLL cohorts [8].

Once an accessible site of greatest FDG avidity is identified, pathology should be obtained by means of an excisional or core biopsy (Figure 1). Falchi et al. had illustrated the importance of adequate tissue sampling for diagnostic classification by performing concurrent fine needle aspiration (FNA) and core or excisional biopsy of the same lesions in 88 patients with suspected RT. Among 53% of the FNA samples, malignant cells were altogether absent or the lesion was histologically downgraded [8]. Therefore, FNA is not recommended.

To accurately discern RT, high-quality biopsy samples must then be reviewed by an expert hematopathologist, with strict adherence to the World Health Organization (WHO) diagnostic criteria [1]. Subsequently establishing the clonal relationship of DLBCL to CLL is imperative given the associated prognostic and therapeutic implications and can be performed by comparing clonal immunoglobulin rearrangement or immunoglobulin heavy-chain variable region gene (IGHV) sequencing [15].

## 4. Survival Outcomes and Prognosis

The reported median survival of patients with RT is 12 months in a large retrospective study [21]. Patients who respond to initial chemotherapy, particularly those who undergo allogeneic hematopoietic stem cell transplant (allo-HSCT), may experience more favorable survival outcomes, with a median post-allo-HSCT survival of 55.4 months [21]. However, for most patients, relapse remains common, emphasizing the urgent need for novel and more effective therapies. The current consensus treatment for RT is chemoimmunotherapy, generally following the treatment algorithm for DLBCL. As outcomes remain poor with this treatment, we recommend challenging this standard. In the next section we describe novel treatment strategies deployed that may hold promise to move the needle in terms of outcomes of patients with RT.

## 5. Novel Strategies and Updates

### 5.1. Targeted Agents and Chemoimmunotherapy (Table 1)

#### 5.1.1. BCL-2 Inhibition with and Without Chemoimmunotherapy

Overexpression of the antiapoptotic protein BCL-2 is a common feature of CLL and RT that supports unrestrained clonal proliferation [22,23]. In the context of CLL, the selective oral BCL-2 inhibitor venetoclax proved to be effective in both the upfront and relapsed or refractory (R/R) settings, subsequently leading to its approval [24].

**Table 1:** Summary of key RT clinical trials and outcomes—Section 5.1.

Trial, Phase	Regimen(s)	Pts, N	Median Survival, Months		Response			Ref.
			OS	PFS	ORR	CR	DoR, Months	
NCT01328626, I	VEN	7	NR	NR	42.9%	0.0%	NR	[24]
NCT03054896, II	VEN + DA-EPOCH-R	26	19.6	10.1	61.5%	50.0%	NR	[25]
NCT03054896, II	VR-CHOP	25	19.5	7.2	68.0%	48.0%	NR	[26]
ACE-CL-001, I/II	Acalabrutinib	25	NR	3.2	40.0%	8.0%	6.2	[27]
BGB-3111-AU-003, I/II	Zanubrutinib	13	29.3	17.3	61.5%	15.4%	25.4	[28]
BRUIN, I/II	Pirtobrutinib	82	12.5	3.7	50.0%	13%	7.4	[29]
BELLWAVE-001, I/II	Nemtabrutinib	6	NR	NR	16.7%	0.0%	NR	[30]

CR: complete response; DA-EPOCH-R: dose-adjusted etoposide, prednisone, vincristine, cyclophosphamide, doxorubicin, and rituximab; DoR: duration of response; NR: not reported; ORR: overall response rate; OS: overall survival; PFS: progression-free survival; Pts: patients; (V)R-CHOP: (venetoclax) + rituximab, cyclophosphamide, doxorubicin, vincristine, prednisone; reference; VEN: venetoclax.

Given its encouraging efficacy in CLL, venetoclax monotherapy and CIT combination strategies have been evaluated in RT. In a phase I study of single-agent venetoclax in RT, partial response (PR) was attained in three of seven patients [24]. A single-arm phase II trial of venetoclax (ramp up to 400 mg, dosed days 22–27 in cycle 1 and days 1–10 in subsequent cycles) plus DA-EPOCH-R (dose-adjusted etoposide, prednisone, vincristine, cyclophosphamide, doxorubicin, and rituximab) demonstrated a notable complete response (CR) rate of 50% (13 of 26), with an overall response rate (ORR) of 62% [25]. Furthermore, participants benefited from relatively durable responses, with a median progression-free survival (PFS) of 10.1 months and median overall survival (OS) of 19.6 months. Importantly, tumor lysis syndrome was not observed, but treatment-emergent adverse events (TEAEs) did include grade ≥3 neutropenia in 65% (including febrile neutropenia in 38%) and thrombocytopenia in 50% of patients [25].

Initial results from a subsequent phase II trial of venetoclax (same dosing schedule) plus R-CHOP, along with mandatory granulocyte colony-stimulating factor (GCSF) support, demonstrated comparable efficacy outcomes with reduced rates of hematologic toxicities to venetoclax + DA-R-EPOCH [26]. This study is ongoing, with preliminary results reported in 25 patients who received a median number of four (range: 1–6) cycles of VR-CHOP, demonstrating high overall (68%) and complete (48%) response rates with fewer events of grade ≥ 3 neutropenia (36%) and only 32% having grade ≥ 3 febrile neutropenia.

A recent multicenter retrospective analysis evaluated the outcomes of patients with RT treated with venetoclax-based regimens (venetoclax plus BTKi, n = 28; R-CHOP, n = 13; other intensive CIT, n = 21) outside of clinical trials [31]. The VR-CHOP cohort experienced encouraging and durable benefits, as evidenced by an ORR of 54%, CR of 46%, and a median PFS and OS of 14.9 months and not reached, respectively. In the overall cohort, the presence of pretreatment del(17p) or *TP53*-mutated CLL was associated with lower CR rates, and there was a trend toward a decreased likelihood of CR among those with prior venetoclax exposure for the treatment of CLL (OR 0.40, 95% CI 0.10–1.63; *p* = 0.33) [31].

Given the findings of these phase II and retrospective studies, we have adopted VR-CHOP as our preferred upfront regimen for RT, as it appears patients in these studies have improved outcomes compared to historical controls.

#### 5.1.2. Covalent BTK Inhibition with and Without Chemoimmunotherapy

Bruton’s tyrosine kinase (BTK) has been implicated in the pathogenesis of B-cell lymphomas through its role in the B-cell receptor (BCR) signaling cascade, providing a strong rationale for its inhibition as a therapeutic strategy [32]. The covalent BTK inhibitors (cBTKis) have transformed the treatment paradigm of CLL, prompting interest in their potential use in RT.

##### Ibrutinib

Ibrutinib is a first-in-class once-daily oral cBTKi [33]. In a retrospective series of four patients with RT (three with R/R RT and one with newly transformed del(17p) CLL), ibrutinib monotherapy led to CR in one and PR in two patients. Despite promising efficacy, the response was not durable with a median duration of response (DoR) of 6.1 months (95% CI 2.8–10.8) [34].

##### Acalabrutinib

The single-arm phase I/II study ACE-CL-001 investigated the selective oral second-generation BTKi acalabrutinib (200 mg twice daily) in patients with RT [27]. Out of 25 patients, the ORR was 40.0%, including CR in two and PR in eight patients, with a median DoR of 6.2 months (95% CI 0.3–14.8). PFS was short, at only 3.2 months (95% CI 1.8–4.0). Interestingly, the ORR was similar among the 11 patients with untreated RT (36% [95% CI 10.9–69.2]) compared to the 14 patients who had received prior treatment (43% [95% CI 17.7–71.1]). In contrast, among the 12 patients with prior ibrutinib exposure, only three (25%) achieved a response [27]. Overall, ACE-CL-001 suggested that acalabrutinib harbors some, albeit limited, single-agent efficacy in RT.

This set the stage for STELLAR, a subsequent randomized phase II study of acalabrutinib (100 mg twice daily, days 6–21) plus standard-of-care R-CHOP (rituximab, cyclophosphamide, doxorubicin, vincristine, and prednisone) versus R-CHOP alone in newly diagnosed RT [35]. Recruitment is ongoing.

##### Zanubrutinib

Zanubrutinib is another potent oral second-generation cBTKi that demonstrated single-agent efficacy in RT. In the BGB-3111-AU-003 open-label phase I/II study (n = 13), zanubrutinib resulted in an ORR of 61.5%, including two (15.4%) cases of CR, with a median DoR of 25.4 months (95% CI 0–29.7) and a median PFS of 17.3 months (95% CI 0.6–32.2) [28]. These responses were higher than previously observed for other cBTKis, which led zanubrutinib to be used in combination with other novel therapies described below.

#### 5.1.3. Non-Covalent BTKis

Nemtabrutinib and pirtobrutinib are reversible, non-covalent BTKis (ncBTKis) that are effective in B-cell malignancies with either wild-type or C481S-mutated BTK. The latter is driven by their distinct mechanism that overcomes the C481S mutation that confers resistance to covalent BTKi [36,37].

##### Pirtobrutinib

Pirtobrutinib is a highly selective BTKi with a long half-life of 19 h, which allows it to exert continuous BTK inhibition [38]. In the open-label phase I/II BRUIN study, single-agent pirtobrutinib was evaluated in a subgroup with RT (n = 82), 90% of whom had received prior RT-directed therapies [29]. An ORR of 50.0% was observed, consisting of CR in 11 (13%) and PR in 30 (37%) patients. Among 18 patients with known clonally related RT, out of 21 patients with available clonality data, the ORR was equal to those patients with clonally unrelated disease at 61.1% (95% CI 35.7–82.7), with three (17%) patients achieving CR. Median DoR, however, was short-lived at 7.4 months (95% CI 3.1–19.1) [29]. Pirtobrutinib is also under investigation for RT in combination with venetoclax and obinutuzumab in an ongoing phase II trial, with results pending (NCT05536349) [39]. Although the DoR for pirtobrutinib was short, it may be an ideal medication to bridge patients to more aggressive therapy, such as anti-CD19 chimeric antigen receptor T-cell therapies (CAR-T) or allo-HSCT.

##### Nemtabrutinib

Nemtabrutinib is a less selective ncBTKi, exerting concurrent inhibitory function on Src kinases, AKT, and ERK [36]. The rationale for its use in RT comes from a preclinical RT-like murine model, in which nemtabrutinib was able to overcome C481S *BTK* and *PLCγ2* mutations and thus increase survival compared to ibrutinib [36]. In the phase I/II BELLWAVE-001 study, nemtabrutinib monotherapy led to PR in one of six patients with RT [30]. While the aforementioned ncBTKis have merit in RT, large prospective studies that directly compare their efficacy and safety to those of existing therapies are necessary in order to evaluate their role in this setting.

#### 5.1.4. Anti-CD79b Antibody Drug Conjugate and Chemoimmunotherapy

Polatuzumab vedotin is an anti-CD79b antibody drug conjugate that has gained FDA approval in combination with R-CHP for untreated DLBCL [40,41]. CD79b was also previously found to be present in 84% of tissue samples derived from 19 patients with RT [42]. An ongoing phase II trial (NCT04679012) is expected to report on polatuzumab in combination with DA-EPOCH-R as upfront therapy in treatment-naïve RT [43].

### 5.2. Combination Treatments with Immunotherapy (Table 2)

#### 5.2.1. BTK Inhibition and Immunotherapy

Combining BTK inhibitors with immunotherapy offers a novel treatment approach in managing RT. PD-1 expression was seen and demonstrated high intensity in patients with RT and a correlation between neoplastic B-cell PD-1 immunohistochemistry positivity and molecularly defined CLL clonal relatedness in RT [44]. This strategy was deployed first in a single-center phase II study administering pembrolizumab to 25 patients (16 with R/R CLL, nine with RT). Only the patients with RT had a response, with one patient achieving a CR (11%), three patients (33%) having a partial metabolic response, four patients (44%) with stable disease, and one patient with progression (11%) [45]. Given preclinical studies showing that BTK inhibition may improve T-cell function [46], a phase II study combining nivolumab with ibrutinib in R/R RT was implemented. The ORR was 42% with a median duration of treatment of 8.4 months for responders compared with 2.6 months for non-responders. The median OS was 13 months, with 25 months for responders vs. 7.6 months for non-responders [47]. Forty-five percent of patients with del(17p) and/or *TP53* mutations had a response. Seven of eleven (64%) patients who were BTKi-naive responded to treatment compared to three out of thirteen (23%) patients who had prior BTKi exposure. In the recent RT1 phase II trial, the combination of zanubrutinib with the PD-1 inhibitor tislelizumab achieved an ORR of 58.3%, with 9 out of 48 patients (18.8%) achieving a CR. The median PFS was 10 months, with median OS not reached and a 12-month OS rate of 74.7% (95% CI 58.4–91.0) [48]. Zanubrutinib plus tislelizumab was recently added to the NCCN guidelines following the publication of these results [17]. Given that the median OS was not reached in this trial, this combination strategy becomes appealing, and we look forward to long-term results.

**Table 2:** Summary of key RT clinical trials and outcomes—Section 5.2.

Trial, Phase	Regimen(s)	Pts, N	Median Survival, Months		Response			Ref.
			OS	PFS	ORR	CR	DoR, Months	
NCT02420912, II	Nivolumab + Ibrutinib	24	25	NR	50%	33%	15	[47]
NCT04271956, II	Tislelizumab + Zanubrutinib	59	NR	10	58.3%	18.8%	NR	[48]
NCT04082897, II	Atezolizumab + Venetoclax + Obinutuzumab	28	64.3	42.9	67.9%	28.6%	NR	[49]

CR: complete response; DoR: duration of response; HDMP: high-dose methylprednisolone; NR: not reported; ORR: overall response rate; OS: overall survival; PFS: progression-free survival; Pts: patients.

#### 5.2.2. BCL-2 Inhibition and Immunotherapy

The MOLTO trial (NCT04082897) explored a chemotherapy-free approach in RT by combining venetoclax, atezolizumab (PD-L1 inhibitor), and obinutuzumab (anti-CD20 antibody). This trial enrolled 28 patients with biopsy-proven RT, achieving a significant ORR of 67.9% with a CR rate of 28.6%. The median PFS was 42.9% at 12 months and OS reached 64.3% at 12 months [49].

In a subgroup analysis, patients with high-risk genetic alterations such as *TP53* mutations had shorter DoRs and survival. Treatment was generally well tolerated, with primary adverse events including neutropenia, infection, and mild immune-related effects. The immunotherapy combination demonstrated sustained reductions in immune-suppressive cells (Tregs and PD-1^+^CD8^+^T-cells), suggesting efficacy both in tumor control and modifying the RT-supportive immune microenvironment [49]. This promising triplet therapy may offer a practical first-line alternative for RT patients, particularly for those who cannot tolerate intensive chemotherapy regimens.

### 5.3. Combination Treatment Using PI3K Inhibitors (Table 3)

#### 5.3.1. PI3Ki with BCL-2is

The basis for dual PI3K-δ/γ and BCL-2 targeting originated from a patient-derived RT xenograft model that demonstrated the synergistic inhibitory effect of duvelisib with venetoclax [50]. When duvelisib induces PI3K inhibition with downstream proteasomal degradation of c-Myc and Mcl-1 in RT cells, simultaneous BCL-2 targeting results in a coordinated antiproliferative and apoptotic response [50]. Among nine patients with RT who were treated with duvelisib plus venetoclax (ramped up to 400 mg daily) as part of a phase I/II study (NCT03534323), CR was achieved in three patients. One patient received allo-HSCT and had a sustained remission measured in years [51]. In another phase I/II trial (NCT03379051), the safety and efficacy of the triple combination of PI3K inhibition (umbralisib) and venetoclax in conjunction with the anti-CD20 antibody ublituximab was examined [52]. In an RT cohort of five patients, two achieved CR while three experienced progressive disease [52].

**Table 3:** Summary of key RT clinical trials and outcomes—Section 5.3.

Trial, Phase	Regimen(s)	Pts, N	Median Survival, Months		Response			Ref.
			OS	PFS	ORR	CR	DoR, Months	
NCT03534323, I/II	Duvelisib + VEN	8	NR	NR	50.0%	37.5%	NR	[51]
NCT03379051, I/II	Umbralisib + VEN + ublituximab	5	NR		40.0%	40.0%	NR	[52]
NCT03884998, I	Copanlisib + nivolumab	14	NR	2.0	33.3%	16.7%	NR	[53]
NCT02332980, II	Pembrolizumab	26	NR	2.6	23.1%	7.7%	NR	[54]
	Pembrolizumab + idelalisib or ibrutinib	16	NR	7.6	62.5%	25.0%	4.5	

CR: complete response; DoR: duration of response; NR: not reported; ORR: overall response rate; OS: overall survival; PFS: progression-free survival; Pts: patients; VEN: venetoclax.

#### 5.3.2. PD-1 Inhibition with PI3Kis

The PD-1 inhibitors pembrolizumab and nivolumab have demonstrated promising efficacy outcomes in RT and when PI3K is concurrently targeted, antitumor effects may be further enhanced [45,47,55]. A phase I investigation (NCT03884998) into the combination of copanlisib plus nivolumab has demonstrated promising outcomes in a heavily pretreated population with R/R RT (n = 14), among whom an ORR was achieved in 31% (including CR in two and PR in two). The median DoR and PFS, in turn, were 15.2 months (95% CI 3.0-NA) and 2.0 months (95% CI 0.7–4.9), respectively [53]. Results from the phase I study of duvelisib plus nivolumab in RT (NCT03892044) are pending [56].

Pembrolizumab has likewise led to encouraging results when combined with a BCR inhibitor—targeting either PI3K with idelalisib or BTK with ibrutinib—in a phase II trial [54]. The initial study phase (n = 26) evaluated pembrolizumab monotherapy, which led to a modest ORR of 23.1%, including CR in two (7.7%) and PR in four (15.4%) after a median of two cycles. Concurrent idelalisib (n = 1) or ibrutinib (n = 15) were added to those who experienced progressive or stable disease on single-agent pembrolizumab, which led to an impressive ORR of 62.5%, including CR in four (25.0%) and PR in six (37.5%) patients after a median of four cycles. The median PFS was 2.6 months (95% CI 1.6–3.6) with pembrolizumab compared to 7.6 months (95% CI 2.3–13.3) in the combination cohort. Twenty-one (81%) patients experienced grade ≥ 3 TEAEs, which most commonly included hematologic toxicities (neutropenia in 38%, thrombocytopenia in 27%, anemia in 15%) and febrile neutropenia (12%) [54].

### 5.4. Bispecific T-Cell Engagers (Table 4)

Bispecific T-cell engagers (BiTEs) are an innovative immunotherapy approach for RT, leveraging targeted T-cell activation to treat aggressive B-cell malignancies. Several BiTEs, particularly epcoritamab, glofitamab, and mosunetuzumab, have shown promising results in clinical studies for RT. Epcoritamab, a CD3/CD20 bispecific antibody, is being studied in RT within the ongoing EPCORE CLL-1 trial (NCT04623541) and has the most data to support its use in RT. Recent updates with 35 patients enrolled showed an ORR of 50% and CR rate of 39%. Cytokine release syndrome (CRS) remained the most frequent TEAE, occurring in 80% of patients [57]. Studies are ongoing, combining glofitamab with polatuzumab vedotin or atezolizumab (NCT06043674), which is a novel strategy. Mosunetuzumab, also a CD3/CD20 bispecific, has shown promising results as a monotherapy in R/R RT, achieving an investigator-assessed ORR and CR rate of 40% and 20%, respectively. The safety profile of mosunetuzumab is notable for manageable CRS (65%, 5% Grade 3), making it a viable chemotherapy-free option for patients with RT, especially those with limited tolerance for more intensive treatments [58].

**Table 4:** Summary of key RT clinical trials and outcomes—Section 5.4.

Trial, Phase	Regimen(s)	Pts, N	Median Survival, Months		Response			Ref.
			OS	PFS	ORR	CR	DoR, Months	
NCT04623541, I/II	Epcoritamab	35	NR	12.8	50%	35%	NR	[57]
NCT02500407, I/II	Mosunetuzumab	20	NR	NR	40%	20%	NR	[58]

CR: complete response; NR: not reported; ORR: overall response rate; OS: overall survival; PFS: progression-free survival; Pts: patients.

### 5.5. CAR T-Cell Therapy (Table 5)

CAR-T has proven to be effective in R/R DLBCL, leading to the FDA approval of three products—axicabtagene ciloleucel (axi-cel), lisocabtagene maraleucel (liso-cel), and tisagenlecleucel (tisa-cel)—in the second- and third-line settings [59,60,61]. In RT, however, experience with CAR-T is more limited.

**Table 5:** Summary of ongoing RT clinical trials—Section 6 and Section 7.

Trial, Phase	Regimen(s)	Pts, N	Median Survival, Months		Response			Ref.
			OS	PFS	ORR	CR	DoR, Months	
STELLAR, II	Acalabrutinib + R-CHOP	Ongoing						[35]
	R-CHOP							
NCT05536349, II	Pirtobrutinib+ VEN + Obinutuzumab	Ongoing						[39]
NCT04679012, II	Polatuzumab + DA-EPOCH-R	Ongoing						[43]
NCT05025800, I/II	Evorpacept + Rituximab + Lenalidomide	Ongoing						[62]
NCT03892044, I	Duvelisib + Nivolumab	Ongoing						[56]
NCT05672173, II	Liso-cel + Nivolumab + Ibrutinib	Ongoing						[63]
NCT05873712, II	Liso-cel + Zanubrutinib	Ongoing						[64]

CR: complete response; DA-EPOCH-R: dose-adjusted etoposide, prednisone, vincristine, cyclophosphamide, doxorubicin, and rituximab; DoR: duration of response; NR: not reported; ORR: overall response rate; OS: overall survival; PFS: progression-free survival; Pts: patients; R-CHOP: rituximab, cyclophosphamide, doxorubicin, vincristine, prednisone; reference; VEN: venetoclax.

An international multicenter retrospective study has recently established the clinical utility of CAR-T in RT [65]. In a cohort of 69 patients who received commercial CAR-T products for RT following heavy pretreatment (including prior BTK and BCL-2 inhibition in 84%), the ORR and CR were 63% and 46%, respectively. Those who achieved CR exhibited durable response, with a median DoR of 27.6 months (95% CI 14.5-NR). Following a median follow-up of 2 years, the median PFS and OS were 4.7 months (95% CI 2.0–6.9) and 8.5 months (95% CI 5.1–25.4), respectively. Grade ≥ 3 TEAEs included neutropenia (87%), thrombocytopenia (71%), CRS (16%), and immune effector cell-associated neurotoxicity syndrome (37%). Furthermore, the study highlighted the significance of infections post-CAR-T, which were documented in 20.3% of cases, while 66.7% experienced febrile neutropenia. Additionally, nine of 12 non-relapse mortality cases were attributed to infections [65].

Two additional retrospective studies have confirmed the efficacy of CAR-T for RT. One compared CAR-T for RT (n = 30) to CAR-T for aggressive B-cell lymphoma (n = 283) and showed similar safety and inferior efficacy outcomes in the RT cohort [66]. At 100 days, patients with RT demonstrated an ORR of 57% and CR of 47%. Additionally, patients with RT experienced worse median (9.9 vs. 18 months) and 12-month (45% vs. 62%) OS compared to DLBCL, with inferior survival associated with increased lines of prior therapy, elevated LDH, and RT histology [66]. In another international retrospective study, the European research initiative on CLL found the ORR to be 65% when CAR-T was used for RT in 54 patients, with 50% of patients attaining CR [67].

These data support the continued use of CAR-T for patients with RT, especially after front-line therapy. Its utility may be improved with a combination approach. As such, multiple phase II trials investigating CAR-T products as mono or combination therapies for RT are underway; these include liso-cel with nivolumab and ibrutinib (NCT05672173) and liso-cel with zanubrutinib (NCT05873712) [63,64].

### 5.6. Hematopoietic Stem Cell Transplantation

Allo-HSCT should be considered for all patients with RT who achieve remission following induction therapy, as studies have shown that allo-HSCT provides a prolonged survival benefit in these patients [68]. Recent analyses underscore that achieving at least PR before transplant is essential, as patients with refractory disease tend to have worse outcomes [68,69]. Outcomes for allo-HSCT in RT depend on the disease status prior to transplant, where patients with a CR had a 3-year PFS and OS of 66% and 77% compared to patients with PR, who had a 3-year PFS and OS of 43% and 57% [70]. However, the high risk of complications such as graft-versus-host disease and transplant-related mortality necessitates a thorough risk–benefit analysis when considering allo-HSCT for patients with RT. While these results are encouraging, most patients with RT are not ideal candidates for allo-HSCT due to inherent chemo-resistant disease and various comorbidities [71]. While the advent of novel agents has generally reduced the need for allo-HSCT in CLL, allo-HSCT remains an essential option for RT, particularly given the high relapse rates associated with standard therapies alone.

## 6. Other Novel Targeted Agents

Alongside the aforementioned emerging therapies for RT, several novel agents have demonstrated promise in early-phase studies (Table 5).

### 6.1. DTRM-555

DTRM-555 is an oral triple combination consisting of the selective cBTKi DTRMWXHS-12 (DTRM-12), everolimus, and pomalidomide. Its applicability for RT was first hypothesized on the basis of preclinical studies that demonstrated an enhanced antitumor effect on a lymphoma xenograft with the addition of an immunomodulatory drug to the already synergistic duo of BTKis with inhibitors of mTOR [72,73]. This culminated in an open-label phase I study (NCT02900716) in R/R lymphomas utilizing a sequential titration design from single-agent DTRM-12 to a doublet with everolimus and a triplet with pomalidomide [74]. Among six patients with RT, one (50%) and two (50%) of those allocated to the doublet and triplet regimens, respectively, achieved PR. In the overall cohort, the triplet patients fared worse than the doublet patient, with a time to initial response of 2.8 (IQR 1.8–3.7) vs. 1.7 months, a median DoR of 4.2 (IQR 1.5–7.0) vs. 11.6 months, and median PFS of 7.0 (IQR 5.2–8.8) vs. 13.3 months. Grade ≥ 3 TEAEs were mostly hematologic, with febrile neutropenia occurring more often with triplet therapy [74]. The phase II trial NCT04305444 is expected to further elucidate the efficacy and safety of DTRM-555 [75].

### 6.2. MK-2140 (Zilovertamab Vedotin)

Receptor tyrosine kinase-like orphan receptor 1 (ROR1) is a transmembrane protein that plays a key role in cellular proliferation in the embryonic stage of life. Its pro-survival properties make it central to tumorigenesis in various malignancies, while its absence in normal tissues make it an appealing cancer-specific target [76,77]. Moreover, preclinical models have shown a correlation between high-level ROR1 expression and both hastened CLL progression and inferior DLBCL-related survival [76,78].

These findings provided rationale for studying zilovertamab vedotin (ZV, otherwise known as MK-2140), a humanized monoclonal anti-ROR1 antibody–drug conjugate, in RT [77]. WaveLINE-001 (NCT03833180) is a phase I trial that evaluated ZV in R/R NHL [79]. Among seven patients with RT treated with ZV, after a median of six (1–10) prior treatment lines, of whom two had previously undergone CAR-T/CAR–natural killer cell therapy, the ORR was 57% (1 CR, 3 PR) with a median DoR of 2.8 (0.0–2.8) months. While ZV was overall tolerable, twenty-seven (48%) of the overall cohort did experience grade 3/4 TEAEs, which most commonly included neutropenia (32%) [79]. WaveLINE-006 (NCT05458297), a phase II study of ZV as a monotherapy and with nemtabrutinib in B-cell malignancies, is recruiting participants for an RT cohort [80,81].

## 7. Future Directions and Emerging Therapies

Recent advancements in targeted therapies and immunotherapy are transforming the treatment landscape for RT, with several promising agents under investigation in early-phase trials aiming to enhance response rates and improve survival outcomes.

1. PRT2527: PRT2527 is a selective and potent cyclin-dependent kinase 9 inhibitor that exhibits antiproliferative and proapoptotic activity in DLBCL, CLL, and mantle cell lymphoma (MCL) cell lines [82]. When combined with a BCL-2 or BTK inhibitor, its antineoplastic properties are potentiated in DLBCL cell line- and patient-derived xenograft models [82]. NCT05665530 is an actively recruiting phase I study that will investigate PRT2527 as a monotherapy and combined with zanubrutinib or venetoclax in R/R hematologic malignancies including RT [83].
2. BGB-16673: A novel BTK-targeted protein degrader, BGB-16673 is under investigation for B-cell malignancies, including RT. By utilizing targeted protein degradation, this agent aims to overcome resistance mechanisms that commonly arise in patients with previous BTK inhibitor exposure [84].
3. LP-118: A novel inhibitor targeting both BCL-2 and B-cell lymphoma extra-large (BCL-XL) proteins, designed to induce apoptosis in B-cell malignancies. Its mechanism is similar to that of venetoclax but has been optimized for greater efficacy, particularly in overcoming resistance observed with venetoclax treatment. Preclinical studies have demonstrated that LP-118 effectively induces cell death in venetoclax-resistant chronic lymphocytic leukemia (CLL) models, suggesting its potential utility in treating RT patients [85]. It is currently in clinical trial (NCT04771572).
4. BGB-21447: another BCL-2 inhibitor, BGB-21447, is being studied specifically in mature B-cell malignancies, including RT (NCT05828589) [86].
5. NX-1607: NX-1607, a first-in-class inhibitor of Casitas B-lineage Lymphoma B (CBL-B), works by enhancing immune cell activation. Early findings suggest that this immune-modulating approach may improve treatment efficacy in RT and other refractory lymphomas, especially when used in combination with standard therapies (NCT05107674) [87,88].

These advancements in targeted therapies and immunotherapies offer new hope for patients with RT, with several agents demonstrating potential efficacy and manageable safety profiles in early trials. As these studies progress, they may redefine the standard of care, particularly for patients who are refractory to conventional treatments.

## 8. Conclusions

While the prognosis for RT remains guarded, ongoing clinical trials and emerging therapies are ushering in new hope. The development of targeted therapies such as BTK-targeting agents, BCL-2 inhibitors, and novel immunotherapies including antibody–drug conjugates, bispecific antibodies, and CAR-T marks a significant step forward. These targeted agents hold the potential not only to improve response rates and survival but also to reduce treatment toxicity for patients with RT, offering a more tailored approach. As these therapies progress through clinical trials, they may soon redefine the treatment paradigm for RT, providing more personalized and effective options for this historically difficult-to-treat disease.
